# Experimental realization and synchronization of a quantum van der Pol oscillator

**DOI:** 10.1126/sciadv.ady5649

**Published:** 2025-10-10

**Authors:** Yi Li, Zihan Xie, Xiaodong Yang, Yue Li, Xingyu Zhao, Xu Cheng, Xinhua Peng, Jun Li, Eric Lutz, Yiheng Lin, Jiangfeng Du

**Affiliations:** ^1^ CAS Key Laboratory of Microscale Magnetic Resonance and School of Physical Sciences, University of Science and Technology of China, Hefei 230026, China.; ^2^ Anhui Province Key Laboratory of Scientific Instrument Development and Application, University of Science and Technology of China, Hefei 230026, China.; ^3^ Hefei National Laboratory, University of Science and Technology of China, Hefei 230088, China.; ^4^ Institute of Quantum Precision Measurement, State Key Laboratory of Radio Frequency Heterogeneous Integration, College of Physics and Optoelectronic Engineering, Shenzhen University, Shenzhen 518060, China.; ^5^ Quantum Science Center of Guangdong-Hong Kong-Macao Greater Bay Area (Guangdong), Shenzhen 518045, China.; ^6^ Institute for Theoretical Physics I, University of Stuttgart, D-70550 Stuttgart, Germany.

## Abstract

Classical self-sustained oscillators, which generate periodic motion without periodic external forcing, are ubiquitous in science and technology. The realization of nonclassical self-oscillators is an important goal of quantum physics. We here present the experimental implementation of a quantum van der Pol oscillator, a paradigmatic autonomous quantum driven-dissipative system with nonlinear damping, using a single trapped atom. We demonstrate the existence of a quantum limit cycle in phase space in the absence of a drive and the occurrence of quantum synchronization when the nonlinear oscillator is externally driven. We additionally show that synchronization can be enhanced with the help of squeezing perpendicular to the direction of the drive and, counterintuitively, linear dissipation. We also observe the bifurcation to a bistable phase-space distribution for large squeezing. Our results pave the way for the exploration of self-sustained quantum oscillators and their application to quantum technology.

## Teaser

A trapped ion realizes a quantum van der Pol oscillator synchronized to an external drive, enhanced via dissipation and squeezing.

## INTRODUCTION

The van der Pol oscillator is a prototypical self-sustained oscillator with nonlinear friction (*1*). Because of its versatility, it has played a central role in the theory of nonlinear oscillations (*2*, *3*), the exploration of chaotic dynamics (*4*, *5*), and the study of synchronization (*6*, *7*). The damping term is such that the van der Pol oscillator is supplied with energy for small amplitudes, while energy is dissipated for large amplitudes. Self-oscillations hence occur in the absence of any external force, and the system asymptotically tends to a limit cycle (*2*, *3*). Self-sustained oscillators differ from ordinary nonlinear systems, since they cannot be analyzed using quasilinear methods. Periodic forcing of the van der Pol oscillator leads to chaotic attractors owing to the nontrivial competition between external cyclic driving and internal autonomous oscillations (*4*, *5*). Its limit cycle can additionally be entrained by a weak periodic signal, inducing synchronized oscillations (*6*, *7*).

Theoretical research has recently focused on the quantum version of the van der Pol oscillator (*8*–*22*), a canonical nonlinear model of driven-dissipative open quantum systems. The tunable interplay of coherent drive and incoherent dissipation in these systems generates complex nonequilibrium evolution and nontrivial steady states (*23*–*25*), which make them interesting for quantum technological applications (*26*). The presence of nonlinearities in general and of self-sustained oscillations in particular further increase the complexity of the dynamics (*16*). The properties of quantum and classical van der Pol oscillators are usually quite similar but may strongly differ in the quantum regime due to the effects of quantum fluctuations and quantum coherence (*8*–*22*). Quantum noise is thus expected to hinder quantum synchronization (*9*), while squeezing (*15*) and dissipation (*18*) are predicted to enhance it. A squeezed drive should also lead to quantum metastability associated with large timescale separations in the dynamics (*20*). Although the van der Pol oscillator (*27*) has been introduced the same year as Schrödinger’s equation (*28*), its quantum implementation has remained elusive.

We here experimentally realize a quantum van der Pol oscillator using a single Ca^+^ ion in a Paul trap (*29*) using reservoir engineering techniques (*30*). We analyze both the undriven and the forced nonlinear quantum oscillator, as well as the influence of squeezing. In the undriven case, we observe the emergence of a quantum limit cycle in phase space by reconstructing the evolution of the Wigner function (*31*, *32*). We further demonstrate the occurrence of stable phase synchronization to an external drive (*8*) and determine the corresponding Arnold tongue diagram (*18*). We also find that synchronization is enhanced by linear dissipation in the deep quantum regime, a behavior that is absent in the classical and quantum domains (*18*). Last, we show that squeezing applied perpendicularly to the drive in phase space induces a bifurcation of the steady-state Wigner function and that it additionally increases phase synchronization (*15*).

## RESULTS

### Experimental system

**Fig. 1.**
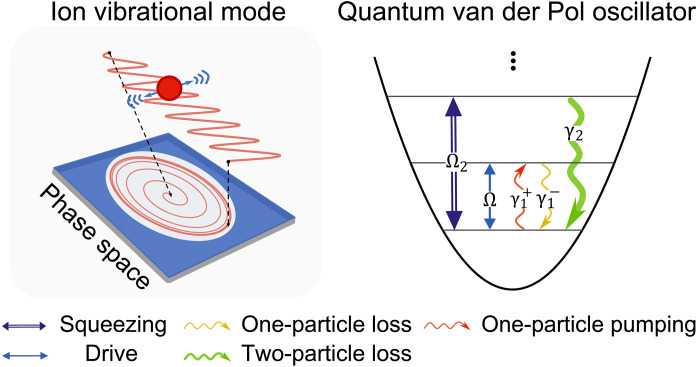
Schematics of the trapped-ion quantum van der Pol oscillator. An ion oscillates around its equilibrium position, forming a vibrational mode subjected to linear and nonlinear dissipation that yields a limit cycle in phase space. The driven-dissipative dynamics is implemented through multiple simultaneous processes: coherent driving with strength Ω , single-particle pumping at rate $\gamma_{1}^{+}$ , single-particle loss at rate $\gamma_{1}^{-}$ (linear damping), two-particle loss at rate $\gamma_{2}$ (nonlinear damping), and, optionally, squeezing with strength $\Omega_{2}$.

We experimentally implement a quantum van der Pol oscillator whose dynamics can be described by the master equation (in units of ℏ ) (*8*–*22*)

$$\dot{\rho }=-i\left[H,\rho \right]+\gamma_{1}^{+}D\left[a^{†}\right]\rho +\gamma_{1}^{-}D\left[a\right]\rho +\gamma_{2}D\left[a^{2}\right]\rho$$ (1)

where $H=-\Delta a^{†}a+i\Omega \left(a^{†}-a\right)+i\Omega_{2}/2\left(a^{†2}e^{2\mathrm{i\theta}}-a^{2}e^{-2\mathrm{i\theta}}\right)$ is the coherent Hamiltonian (with detuning Δ ), including the displacement drive (with strength Ω ) and squeezing drive in the rotating frame (with amplitude $\Omega_{2}$ ); the parameter θ is the relative phase between the two drives. The operators a and $a^{†}$ are the usual ladder operators of the harmonic oscillator, and the Lindblad dissipators are given by $D\left[O\right]\rho =O\rho O^{†}-\left\{\;O^{†}O,\rho \;\right\}\;/\;2$ . The two coefficients $\gamma_{1}^{+}$ and $\gamma_{1}^{-}$ respectively represent the one-particle pumping rate and the one-particle loss rate (associated with linear damping), while $\gamma_{2}$ is the two-particle loss rate (corresponding to nonlinear damping) (Fig. 1). This model is also (more accurately) referred to as the quantum Rayleigh–van der Pol oscillator (*21*, *22*). Equation 1 differs from recent trapped-ion realizations of self-excited phonon lasers (*33*, *34*) through the presence of nonlinear friction. It exhibits distinct regimes depending on the relative dissipation rates: semiclassical for $\gamma_{2}\;/\;\gamma_{1}^{+}≲0.1$ , quantum for $\gamma_{2}\;/\;\gamma_{1}^{+}∼1$ , and deep quantum for $\gamma_{2}\;/\;\gamma_{1}^{+}≳10$ (*18*). Our study primarily focuses on the latter two, where the phonon population remains low and the system displays nonclassical properties.

The experiment uses the axial motion of a single ^40^Ca^+^ trapped ion as the harmonic oscillator with a trapping frequency $\omega_{m}\;\approx \;\left(2\pi \right)\times 1.1$ MHz. The internal spin states are defined as $|\;↓\;\rangle =|S_{1/2},m_{J}=+1\;/\;2\rangle$ , $|\;↑\;\rangle =|D_{5/2},m_{J}=+1\;/\;2\rangle$ , and $|\;\text{aux}\rangle =|D_{5/2},m_{J}=+5\;/\;2\rangle$ to assist laser-induced spin-motion couplings with a narrow-linewidth 729-nm laser along the axial direction. The displacement drive in Eq. 1 is achieved through a radio frequency (rf) signal at frequency $\omega =\omega_{m}+\Delta$ applied to one of the trap electrodes (*35*). Linear and nonlinear damping processes are further realized by reservoir engineering methods (*30*), which use coherent red and blue sideband drives combined with spin resets, coupling the oscillator to the spin such that ∣↓,n〉→∣aux,n±1〉 and ∣↓,n〉→∣aux,n−2〉 , with the spin optically pumped back to ∣↓〉 , effectively forming the motional dissipation with respective strengths of $\gamma_{1}^{\pm }$ and $\gamma_{2}$ (see Materials and Methods for details). We additionally implement squeezing with controllable strength by applying two pairs of laser tones off-resonantly driving the spin-motion joint transition (*36*, *37*). After Doppler and ground-state cooling of the motion (*29*), the displacement drive is then applied continuously, while other coupling and pumping lasers are applied sequentially via a first-order Trotterization approach (*38*). Subsequently, we investigate the properties of the quantum van der Pol oscillator via tomographic state reconstruction that maps the oscillator’s motional information onto spin populations and thus directly measures the Wigner function in polar coordinates W(r,ϕ) (*31*).

### Quantum limit cycle and synchronization

**Fig. 2.**
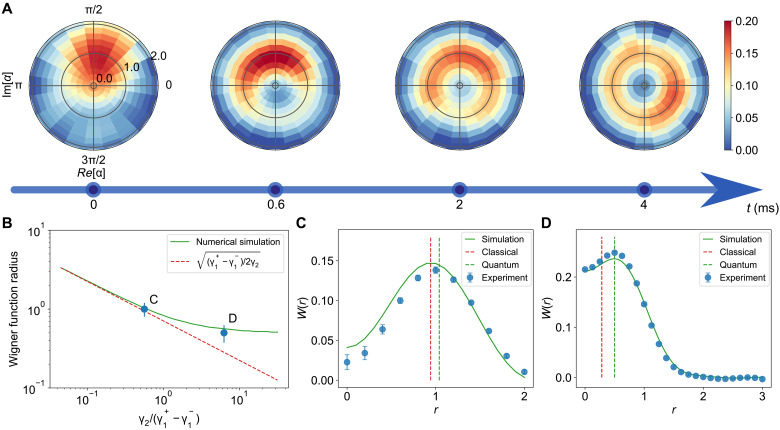
Limit cycle of the undriven quantum van der Pol oscillator. (**A**) Dynamical evolution starting from a displaced thermal state ( $\bar{n}=1.5,\alpha =1$ ). The reconstructed Wigner function W(r,ϕ) evolves into a ring-shape steady-state phase-space distribution, associated with a limit cycle. The dissipative ratio is $\gamma_{2}/\left(\gamma_{1}^{+}-\gamma_{1}^{-}\right)=0.56$ . (**B**) Steady-state radius of the undriven van der Pol oscillator as a function of the dissipative ratio $\gamma_{2}\;/\;\left(\gamma_{1}^{+}-\gamma_{1}^{-}\right)$ . The numerical simulations (green line), experimental results [blue dots, corresponding to (C) and (D)], and the mean-field prediction $\sqrt{\left(\gamma_{1}^{+}-\gamma_{1}^{-}\right)\;/\;2\gamma_{2}}$ (red dashed line) are shown for comparison. (**C** and **D**) Radial Wigner function *W*(*r*) for respective dissipative parameters of $\gamma_{2}\;/\;\left(\gamma_{1}^{+}-\gamma_{1}^{-}\right)=0.56$ and $\gamma_{2}\;/\;\left(\gamma_{1}^{+}-\gamma_{1}^{-}\right)=6.25$ . In the latter case, a larger radius is observed compared with the classical mean-field prediction, highlighting the nonclassical feature. The values of the expected radii of the Wigner function in the numerical simulation and the classical model are indicated with green and red dashed lines, respectively.

Let us begin by analyzing the dynamics of the undriven van der Pol oscillator, where $\Delta =\Omega =\Omega_{2}=0$ . To that end, we prepare the oscillator in a displaced thermal state (*39*), that is, a thermal state with mean phonon number $\bar{n}=1.5$ displaced by a coherent state with amplitude α=1.0 , and measure the Wigner function W(r,ϕ) at times 0, 600, 2000, and 4000 μs for $\gamma_{2}/\left(\gamma_{1}^{+}-\gamma_{1}^{-}\right)=0.56$ (Fig. 2A), with $\gamma_{1}^{\left\{+,-\right\}}=\left\{2.06\;\text{kHz},\;0.09\;\text{kHz}\right\}$ . We observe that the initially localized state first relaxes to a lower amplitude before spreading in phase space to form the typical ring shape of a limit cycle (*8*). This establishes the autonomous nature of the oscillations. The radius of the quantum limit cycle, defined as the position of the maximum of the Wigner function, depends on the dissipation ratio $\gamma_{2}\;/\;\left(\gamma_{1}^{+}-\gamma_{1}^{-}\right)$ (Fig. 2B). It approaches the classical mean-field prediction, $\sqrt{\left(\gamma_{1}^{+}-\gamma_{1}^{-}\right)/2\gamma_{2}}$ (red dashed line) (*18*), for small $\gamma_{2}/\left(\gamma_{1}^{+}-\gamma_{1}^{-}\right)$ . However, contrary to the latter quantity, it does not vanish for large dissipation ratio due to the fact that the first excited Fock state ∣1〉 is immune to the nonlinear dissipation induced by $a^{2}$ , thereby preventing complete occupation of the ground state ∣0〉 (*18*). The radial Wigner function *W*(*r*) is shown in Fig. 2 (C and D) for $\gamma_{2}/\left(\gamma_{1}^{+}-\gamma_{1}^{-}\right)=0.56$ and $\gamma_{2}/\left(\gamma_{1}^{+}-\gamma_{1}^{-}\right)=6.25$ , which agree well with numerical simulations. In the latter case, the quantum radius (green dashed line) deviates from the classical mean-field value (red dashed line), highlighting the nonclassical features of the oscillator.

**Fig. 3.**
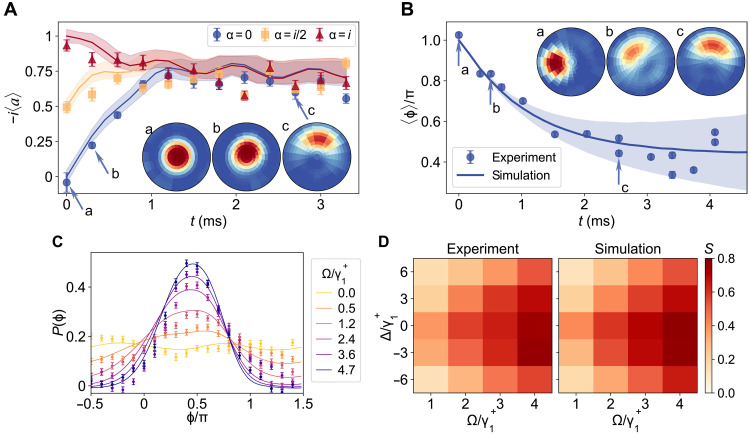
Phase synchronization of the driven quantum van der Pol oscillator. (**A**) The mean amplitude 〈a〉 for different initial coherent states ∣α〉 ( α=0,i/2,i ), with damping and driving parameters $\gamma_{2}\;/\;\gamma_{1}^{+}=5.2$ and $\Omega \;/\;\gamma_{1}^{+}=3.5$ , converges to the same steady oscillations, demonstrating entrainment between oscillator and drive. Insets (a to c) show the evolution of the Wigner function W(r,ϕ) , starting from the vacuum state. The shaded areas are statistical error. (**B**) The mean phase 〈ϕ〉 for an initial coherent state α=−1 , with $\gamma_{2}\;/\;\gamma_{1}^{+}=5.7$ and $\Omega \;/\;\gamma_{1}^{+}=4.7$ , locks to a constant value that slightly differs from the driving direction because of off-resonant effects. The shaded blue area indicates phase fluctuations due to experimental imperfections. Insets (a to c) depict the evolution of the Wigner function, starting from this coherent state. (**C**) The steady-state phase distribution P(ϕ) is a Gaussian centered on the driving phase, whose width decreases with increasing driving strength. Parameters are $\gamma_{2}/\gamma_{1}^{+}=5.7$ and $\Omega \;/\;\gamma_{1}^{+}=\left\{\;0,0.47,1.2,2.4,3.6,4.7\;\right\}$ . (**D**) Arnold tongue for the mean resultant length S as a function of detuning and strength of the driving, defining the synchronization region. The dissipative rate is $\gamma_{2}\;/\;\gamma_{1}^{+}=4.7$ . In all subfigures, solid lines are simulation results.

We next switch on the resonant coherent drive with amplitude Ω according to Eq. 1, oriented along the π/2 direction ( $\Delta =\Omega_{2}=0$ ). The temporal evolution of the average amplitude 〈a〉 of the oscillator is measured for various initial coherent states ∣α〉 , with amplitude α=0,i/2,i , for $\gamma_{2}\;/\;\gamma_{1}^{+}=5.2$ and $\Omega /\gamma_{1}^{+}=3.5$ (Fig. 3A). For all these initial conditions, the mean amplitude 〈a〉 converges to the same steady oscillations, as the van der Pol oscillator adjusts itself to the external drive, indicating entrainment between the two (*1*). The inset further shows that the drive breaks the radial symmetry of the undriven limit cycle, aligning the oscillator’s phase-space distribution with the drive axis. The amplitude is determined by applying spin-dependent forces and fitting the resulting spin dynamics. Because of a finite number of experimental repetitions, statistical fluctuations introduce uncertainties, which are represented as shaded areas in the figure. Additional insight is obtained by examining the phase dynamics of an initial coherent state ∣α〉 with a phase offset relative to the drive α=−1 (Fig. 3B): The mean phase 〈ϕ〉 locks to a constant value close to π/2 (Fig. 3B), a signature of phase synchronization (*8*). The shaded blue area accounts for experimental imperfections, including oscillator frequency and laser frequency fluctuations. Experimental results align closely with numerical simulations (solid lines), with minor deviations (e.g., a slight phase misalignment) attributable to off-resonance effects caused by the sideband transitions (see the Supplementary Materials for details). Figure 3C moreover reveals that the phase distribution, P(ϕ)=∫W(r,ϕ)rdr , adopts a Gaussian profile, centered at the drive phase, that becomes narrower with increasing driving strength $\Omega /\gamma_{1}^{+}$ ( $\gamma_{2}\;/\;\gamma_{1}^{+}=5.7$).

To quantify phase synchronization, we use the mean resultant length of a circular distribution, $S=|\langle e^{\mathrm{i\phi}}\rangle |=|\int_{\phi }e^{\mathrm{i\phi}}P\left(\phi \right)\mathrm{d\phi}|$ (*18*). It takes the value 0 for an unsynchronized state and the value 1 for a perfectly synchronized state, which corresponds to a delta function like phase distribution. We examine the synchronization parameter S as a function of driving strength Ω and detuning Δ , while keeping the dissipative rate fixed at $\gamma_{2}/\gamma_{1}^{+}=4.7$ (Fig. 3D). We find that synchronization increases with the driving strength and decreases with the detuning, as expected. We recognize a structure that is reminiscent of an Arnold tongue, which defines the synchronized domain of classical synchronization phenomena (*6*, *7*). Figure 3D shows that phase locking of the quantum van der Pol oscillator is a robust phenomenon that appears in a large region of parameter space. We again have good agreement with numerical simulations. As before, there is a slight asymmetry in the diagram due to experiment imperfections.

### Quantum dissipation boost and squeezing

**Fig. 4.**
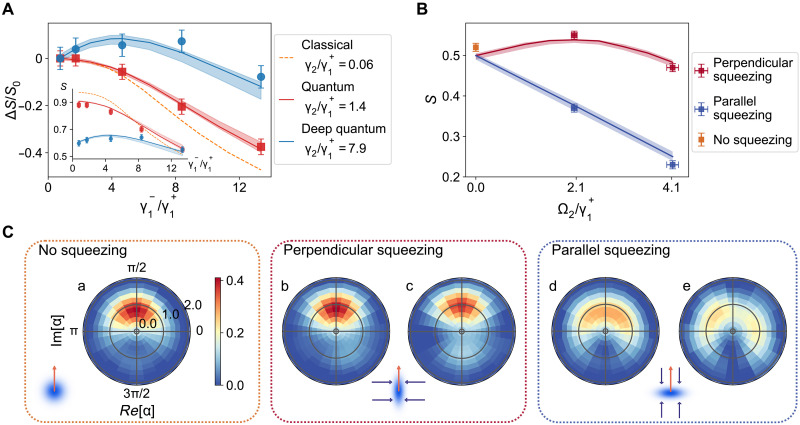
Quantum synchronization enhancement under dissipation and squeezing. (**A**) The enhanced mean resultant length S compared with no single-photon dissipation case as a function of dissipation rate. Moderate single-phonon dissipation increases synchronization in the deep quantum regime ( $\gamma_{2}\;/\;\gamma_{1}^{+}=7.9$ ), but not in the quantum ( $\gamma_{2}\;/\;\gamma_{1}^{+}=1.4$ ) and semiclassical ( $\gamma_{2}\;/\;\gamma_{1}^{+}=0.06$ ) domains. The inset figure shows the raw synchronization strength S with the same *x* axis. Synchronization is consistently stronger in the quantum regime compared to the deep quantum regime, as quantum noise is lower in the former. The external drive is resonant with the oscillator frequency, with a strength of $\Omega \;/\;\gamma_{1}^{+}=3$ . (**B**) Moderate squeezing perpendicular to the drive ( θ=π/2 ) enhances synchronization by suppressing quantum fluctuations, whereas squeezing parallel to the drive ( θ=0 ) decreases it; parameters are $\gamma_{2}\;/\;\gamma_{1}^{+}=4.4$ and $\Omega \;/\;\gamma_{1}^{+}=1.2$ . (**C**) Corresponding Wigner functions to (B): (a) no squeezing $\Omega_{2}=0$ serves as a reference, while (b) and (d) and (c) and (e) correspond to squeezing strengths of $\Omega_{2}\;/\;\gamma_{1}^{+}=2.1$ and $\Omega_{2}\;/\;\gamma_{1}^{+}=4.1$ , respectively. In the schematics, blue arrows indicate the squeezing direction, whereas orange arrows represent the external drive direction. Strong parallel squeezing leads to a bifurcation to a bistable Wigner function (e).

Quantum noise is usually expected to be detrimental for synchronization, as it provides an additional source of phase diffusion (*9*). It is therefore essential to identify quantum mechanisms that allow one to boost synchronization in the quantum realm. We first investigate the influence of single-phonon dissipation by increasing the parameter $\gamma_{1}^{-}$ in three different regimes defined by the strength of the nonlinear damping (*18*): $\gamma_{2}/\gamma_{1}^{+}=7.9$ (deep quantum regime), $\gamma_{2}/\gamma_{1}^{+}=1.4$ (quantum regime), and $\gamma_{2}/\gamma_{1}^{+}=0.06$ (semiclassical regime). The external drive is resonant with the oscillator’s frequency, with a strength of $\Omega /\gamma_{1}^{+}=3$ . Defining the synchronization value near the $\gamma_{1}^{-}\to 0$ point as $S_{0}$ , we compare this with other situations to determine the enhancement of synchronization $\Delta S=S-S_{0}$ . While the inset of Fig. 4A shows that the synchronization is stronger in the classical regime, the main figure illustrates that increasing single-phonon dissipation enhances synchronization ( ΔS>0 ) in the deep quantum regime (blue dots) but reduces it in the quantum (red squares) and semiclassical (yellow dashed line) regimes; the small $\gamma_{2}$ domain is not accessible experimentally and hence simulated. This marked difference can be explained by noting that the decoherence rate grows with the phonon number (*18*), meaning that higher levels decohere faster. By driving the oscillator to lower levels, single-phonon dissipation thus initially helps increase the transition rate between the two lowest levels, that is, build up coherence between ∣0〉 and ∣1〉 , which, in turn, augments synchronization. Larger single-phonon dissipation suppresses quantum synchronization ( ΔS<0 ) in all regimes, as dissipation-induced decoherence becomes dominant.

Another strategy to counteract the negative influence of quantum noise and augment synchronization is squeezing (*15*). Squeezing is able to suppress fluctuations in one direction, at the expense of the orthogonal direction. Figure 4C shows the reconstructed Wigner function for three values of the squeezing strength ( $\Omega_{2}/\gamma_{1}^{+}=\left\{0,2.06,4.12\right\}$ ), when the squeezing direction is either parallel ( θ=π/2 ) or perpendicular ( θ=0 ) to the direction of the displacement drive; the dissipative parameter is $\gamma_{2}/\gamma_{1}^{+}=4.4$ , and the resonant driving strength is $\Omega /\gamma_{1}^{+}=1.2$ . The shape of the Wigner function results from the competition between the displacement drive and the squeezing drive. For small squeezing strengths, the Wigner function remains primarily aligned with the displacement direction. However, as the squeezing amplitude increases and exceeds the displacement strength, the Wigner function splits, signaling the bifurcation from a monostable distribution to a bistable distribution (*20*). Small squeezing perpendicular to the drive ( θ=0 ) reduces the phase variance, thus enhancing synchronization, as seen in the increase of the mean resultant length S , which is evaluated from the Wigner function, in Fig. 4B; this moderate effect, which is statistically significant (exceeding 2σ ), could be further improved with stronger driving and squeezing strength (see the Supplementary Materials for details). By contrast, small squeezing parallel to the drive ( θ=π/2 ) increases the phase variance, which hinders synchronization. In both instances, a larger squeezing parameter, beyond the bifurcation to multistability, reduces the mean resultant length S , beyond the level achieved without squeezing. One may hence conclude that there is an optimal parameter window in which squeezing has a positive effect on quantum synchronization, for instance, shown in Fig. 4 [B and C (b)].

## DISCUSSION

Driven-dissipative quantum systems are substantially influenced by the dissipative part of the dynamics, rather than by their Hamiltonian, and therefore exhibit properties markedly different from those of closed systems at equilibrium. By engineering nonlinear dissipation, we have demonstrated the experimental realization of a quantum van der Pol oscillator using a single trapped ion. Our results offer key insights into quantum limit cycles, phase synchronization, and the interplay between coherence, dissipation, and squeezing. They thus provide a valuable platform to investigate the intricate dynamics of quantum self-sustained oscillators. They could, for example, be harnessed for quantum sensing due to their strong nonlinear response close to criticality (*17*), for quantum computation through dissipation-engineering of robust steady states (*26*, *40*), and for neuromorphic quantum information processing due to enlarged Hilbert space dimension (*41*, *42*). Moreover, extensions to networks of nonlinear oscillators could reveal previously unidentified collective phenomena and dissipative phase transitions, including chimera states and topological phases (*43*–*45*). These systems thus hold immense potential for advancing quantum technologies and deepening our understanding of nonequilibrium quantum dynamics.

## MATERIALS AND METHODS

### Experimental scheme

We demonstrate the quantum van der Pol oscillator through the Trotterization approach (*38*): The target evolution is divided into N steps, each consisting of coherent operations $e^{{\mathrm{iH}}_{j}t/N}$ and dissipative operations $e^{K_{j}t/N}$ , implemented alternately. The total operations can then mimic the original process: ${\text{lim}}_{N\to \infty }{\left(\Pi_{j}e^{{\mathrm{iH}}_{j}t/N}e^{K_{j}t/N}\right)}^{N}=e^{\sum_{j}\left({\mathrm{iH}}_{j}+K_{j}\right)t}$ . The schematic of the circuit is shown in fig. S2.

For the coherent part, an rf signal with frequency $\omega =\omega_{m}+\delta$ is applied to one of the electrodes to generate the displacement driving (*35*). This is the only component always applied during the sequence; other parts are implemented sequentially. We introduce phonon squeezing along the controllable direction and strength by laser driving two noncommuting spin-dependent forces (*37*).

For the dissipative part, the internal spins are introduced as the dissipative channel to engineer the desired jump operators D[O] through sideband transitions and spin reset. Since the three dissipation channels, including $D\left[a^{†}\right]$ , D[a] , and $D\left[a^{2}\right]$ , undergo the similar operation, we only take D[a] as an example. Considering the spin-phonon system whose initial state is ρ⊗∣↓〉〈↓∣ , the red sideband Hamiltonian is

$$H_{\text{rsb}}=\Omega_{\text{rsb}}/2\left(\sigma^{+}a+\sigma^{-}a^{†}\right)$$ (2)

where $\Omega_{\text{rsb}}$ is the Rabi frequency of the first-order red sideband. After sideband transition time $\tau_{\text{rsb}}$ , the density matrix for the oscillator is

$$\begin{array}{c} \begin{array}{c} \rho \left(t+\tau_{\text{rsb}}\right)={\mathrm{Tr}}_{\text{spin}}\left[e^{-\mathrm{iH}\tau_{\text{rsb}}}\rho ⊗|\;↓\rangle \langle \;↓|e^{\mathrm{iH}\tau_{\text{rsb}}}\right]\\ =\langle \;↑|\rho_{\text{tot}}\left(t+\tau_{\text{rsb}}\right)|\;↑\rangle +\langle \;↓|\rho_{\text{tot}}\left(t+\tau_{\text{rsb}}\right)|\;↓\rangle \\ \equiv \rho_{u}+\rho_{d} \end{array} \end{array}$$ (3)

For small times $\tau_{\text{rsb}}$ , we can expand the unitary operators as $e^{\pm \mathrm{iH}\tau_{\text{rsb}}}=1\pm \mathrm{iH}\tau_{\text{rsb}}+o\left(\tau_{\text{rsb}}^{2}\right)$ and obtain

$$\begin{array}{c} \begin{array}{ll} \rho_{u} & \approx \Gamma \tau_{\text{rsb}}\mathrm{a\rho}\left(t\right)a^{†}\\ \rho_{d} & \approx \rho \left(t\right)-\frac{\Gamma }{2}\tau_{\text{rsb}}\left[a^{†}\mathrm{a\rho}\left(t\right)+\rho \left(t\right)a^{†}a\right]+o\left(\tau_{\text{rsb}}^{4}\right) \end{array} \end{array}$$ (4)

where $\Gamma ={\left(\frac{\Omega_{\text{rsb}}}{2}\right)}^{2}\tau_{\text{rsb}}$ . Here, the first-order term $o\left(\tau_{\text{rsb}}\right)$ vanishes, so the second-order expansion is required. After the spin reset, the density matrix of the oscillator would become $\rho =\rho_{u}+\rho_{d}$ , which undergoes the same trajectory as D[a] with effective pumping rate Γ.

## Supplementary Materials

### This PDF file includes:

Supplementary Text Figs. S1 and S2 Tables S1 to S3 References
