# Supplementary material for: Experimental realization and synchronization of a quantum van der Pol oscillator
Source: Sci Adv. 2025 Oct 10;11(41):eady5649. doi: 10.1126/sciadv.ady5649 (PMC12513414; doi:10.1126/sciadv.ady5649)
Supplement: Supplementary file 1 — Supplementary Text Figs. S1 and S2 Tables S1 to S3 References [file sciadv.ady5649_sm.pdf]

Science Advances
AAAS

Supplementary Materials for

Experimental realization and synchronization of a quantum van der Pol oscillator

Yi Li *et al.*

Corresponding author: Xiaodong Yang, yangxd@szu.edu.cn; Jun Li, lijunquantum@szu.edu.cn; Eric Lutz, eric.lutz@itp1.uni-stuttgart.de; Yiheng Lin, yiheng@ustc.edu.cn; Jiangfeng Du, djf@ustc.edu.cn

Sci. Adv. **11**, eady5649 (2025)
DOI: 10.1126/sciadv.ady5649

This PDF file includes:

Supplementary Text
Figs. S1 and S2
Tables S1 to S3
References

Supplementary Text

Mean-field result for the limit cycle of an undriven quantum van der Pol oscillator

Here we derive the radius of the limit cycle for an undriven quantum van der Pol oscillator, using both the mean-field approach and numerical calculation. The Hamiltonian for the oscillator is

$$\dot{\rho} = -i[H, \rho] + \gamma_1^+ \mathcal{D}[a^\dagger]\rho + \gamma_1^- \mathcal{D}[a]\rho + \gamma_2 \mathcal{D}[a^2]\rho. \tag{S1}$$

For simplicity, we work in the rotating frame where $H = 0$ ($\Omega = \Omega_2 = 0, \Delta = 0$). The evolution equation for the expectation value $\langle a \rangle$ can be expressed as

$$\frac{d\langle a \rangle}{dt} = \frac{\gamma_1^+ - \gamma_1^-}{2}\langle a \rangle - \gamma_2 \langle a^\dagger a a \rangle. \tag{S2}$$

In the classical limit $\langle a^\dagger a \rangle \gg 1$, we can replace $\langle a \rangle$ with a complex number α and get

$$\frac{d\alpha}{dt} = \frac{\gamma_1^+ - \gamma_1^-}{2}\alpha - \gamma_2 |\alpha|^2 \alpha, \tag{S3}$$

By letting $\frac{d\alpha}{dt} = 0$, it is evident that the steady state amplitude is $|\alpha| = \sqrt{\frac{\gamma_1^+ - \gamma_1^-}{2\gamma_2}}$.

Numerical simulation of squeezing enhanced synchronization

In the main text, we observe a modest enhancement of synchronization due to squeezing, which is limited by current experimental conditions. To further support this observation, we carry out numerical simulations, as shown in Fig. S1, using a larger drive strength ($\Omega/\gamma_1^+ = 6.8$) to better access the parameter regime where squeezing has a more significant effect. These simulations indicate that the enhancement becomes more pronounced in the deep quantum regime and under optimal squeezing strength. At the same time, we find that the maximum achievable synchronization strength tends to be larger in a less quantum regime. Together, these results confirm the underlying mechanism and illustrate the trade-off between the relative enhancement and raw synchronization strength.

Experimental apparatus and procedure

In this work, we use a single trapped $^{40}Ca^+$ ion in a linear Paul trap (*29*). The quadrupole transition between $|S_{1/2}\rangle$ and $|D_{5/2}\rangle$ is used for coherent manipulations, which is driven by a narrow-linewidth 729 nm laser along the axial direction. The Lamb-Dicke parameter for the spin-motion coupling is $\eta = 0.0925$. As the experiment is performed mostly in the (deep) quantum regime, the phonon is at most excited to 4, which means that the experiments can be well described by the Lamb-Dicke approximation (*29*). The internal electronic state of the ion is initialized to $|\downarrow\rangle$ by the frequency-resolved 729 nm laser combined with 854 nm and 866 nm repump lasers. We use the 397 nm cycling transition between $|S_{1/2}\rangle$ and $|P_{1/2}\rangle$ along with 866 nm repump laser for fluorescence detection.

The axial mode is used to simulate the van der Pol oscillator. Its frequency is measured by "tickle" spectroscopy, where we apply the radio frequency to one electrode and detect the resulting phonon population using the red sideband. We calibrate it every 30 minutes to ensure the long-term stability of the mode frequency within 30 Hz.

Before each experiment, the motional mode is initialized by Doppler cooling and sideband cooling. After sideband cooling, the mean phonon number of the mode can be cooled to less than 0.1. The subsequent experimental procedure involves phonon state preparation (if necessary), dynamic evolution and mode measurement. We prepare the coherent thermal state using an electronic displacement drive, combined with Trotterizations involving blue and red sidebands to simulate the heating process. During the dynamic evolution process, the external drive remains on (except for Fig. 2) throughout the entire evolution. The other operations are segmented. In each cycle, we sequentially apply squeezing (if necessary), one-particle pumping, one-particle loss (if necessary), two-particle loss, and spin state preparation. The state preparation is added to avoid the leakage to $|S_{1/2}, m_J = -1/2\rangle$. The phonon tomography is done right after the evolution. The whole procedure is shown in Fig. S2B and on Tab. S1. Key effective intensity parameters for the experiments are listed in Tab. S2. The pulse time and number of cycles are shown in Tab. S3.

Numerical Simulations and Experimental Imperfections

We simulate the experimental sequence by modeling a two-level system coupled to a phonon mode truncated at 30 excitations. We use the full Hamiltonian instead of a simple linear expansion for

the interaction between spin and motion. As will be discussed below, this gives rise to the motion phase shift caused by the off-resonant coupling of the sideband transitions. These simulations are performed using the QuTip toolbox (*46*). The simulation lines shown in the main text all consider the real experimental issues, including heating rate of phonon and full Hamiltonian of spin-motion couplings. The other imperfections are mainly due to the trapping frequency fluctuation, which is shown as the shadow region in the figures.

Another key experimental issue is the unavoidable off-resonant coupling effect caused the second red sideband transition, which is used in conjunction with spin pumping to implement the two-phonon dissipator $\mathcal{D}[a^2]$. Due to the small Lamb-Dicke parameter, additional coupling to the first red sideband and carrier cannot be neglected. A second-order Magnus expansion shows that this off-resonant interaction generates an effective phonon AC Stark shift, $(\eta\Omega_{2rsb})^2/(2\omega_z)\sigma_z a^\dagger a$, causing a slight shift in the steady-state phase relative to the external drive and minor oscillations during evolution. Nevertheless, these off-resonant effects have only a minimal impact on the mean resultant length compared to the ideal case. In addition, the heating rate of the mode is around 80-150 phonon/s (drift from month to month), which is modeled as jump operators $\sqrt{\gamma_h}a$ and $\sqrt{\gamma_h}a^\dagger$.

The Hamiltonians for the numerical simulation in QuTip are provided below. Motion heating is modeled using this master equation:

$$\dot{\rho} = -i\left[H, \rho\right] + \gamma_h \mathcal{D}\left[a^\dagger\right]\rho + \gamma_h \mathcal{D}\left[a\right]\rho. \tag{S4}$$

The Hamiltonian H includes $H_m = \omega_z a^\dagger a$, displacement drive $H_{dis} = i\Omega a^\dagger e^{-i((\omega_z+\Delta)t+\phi_{dis})} + h.c.$ and spin-motion coupling Hamiltonians, which are listed below, with $e^{ikx} = e^{i\eta(a+a^\dagger)}$.

First-order blue sideband:

$$H_{bsb} = \frac{\Omega_{bsb}}{2\eta}\left[\sigma_+ e^{ikx} e^{-i(\delta_{bsb}t+\phi_{bsb})} + h.c.\right], \tag{S5}$$

First-order red sideband:

$$H_{rsb} = \frac{\Omega_{rsb}}{2\eta}\left[\sigma_+ e^{ikx} e^{-i(\delta_{rsb}t+\phi_{rsb})} + h.c.\right], \tag{S6}$$

Second-order red sideband:

$$H_{2rsb} = \frac{\Omega_{2rsb}}{\eta^2}\left[\sigma_+ e^{ikx} e^{-i(\delta_{2rsb}t+\phi_{2rsb})} + h.c.\right], \tag{S7}$$

Squeezing $H_{sq} = H_{sq,r_+} + H_{sq,r_-} + H_{sq,b_+} + H_{sq,b_-}$:

$$\begin{aligned} H_{sq,r_\pm} &= \frac{\Omega_{sq}}{2\eta} \left\{ \sigma_+ e^{ikx} e^{-i\left[(-\omega_z \pm \delta_m)t + \phi_{sq,r_\pm}\right]} + h.c. \right\}, \\ H_{sq,b_\pm} &= \frac{\Omega_{sq}}{2\eta} \left\{ \sigma_+ e^{ikx} e^{-i\left[(\omega_z \pm \delta_m)t + \phi_{sq,b_\pm}\right]} + h.c. \right\}. \end{aligned} \tag{S8}$$

There are also experimental imperfections in phonon measurements. To construct the Wigner function, we follow the method in Ref. (*31*), which first uses the spin-dependent forces to measure the characteristic function $\chi(r, \phi)$ and then performs the Fourier transformation to obtain the Wigner function $W(r, \phi)$. Due to finite sampling points, the Fourier transformation is not perfect, which leads to potential misalignment between the real Wigner function value and the reconstructed one, especially for the state with a large average phonon number. However, we find such an effect is negligible since the resultant error for for S is around 0.02, on the same level as the statistical error.

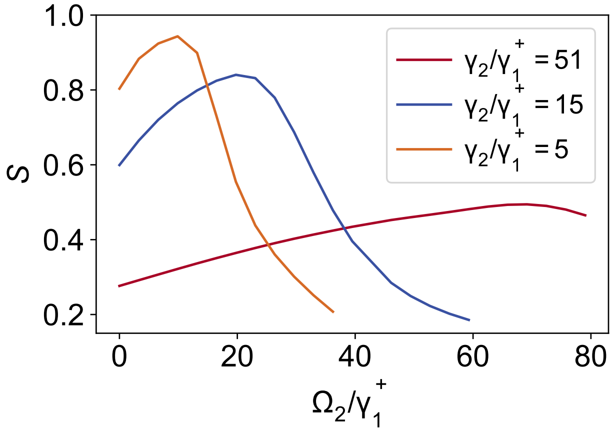

Figure S1: **Squeezing enhanced synchronization under different regimes.** While the enhancement of synchronization becomes more pronounced in the deep quantum regime and under optimal squeezing strength, the maximum achievable synchronization strength tends to be larger in a less quantum regime.

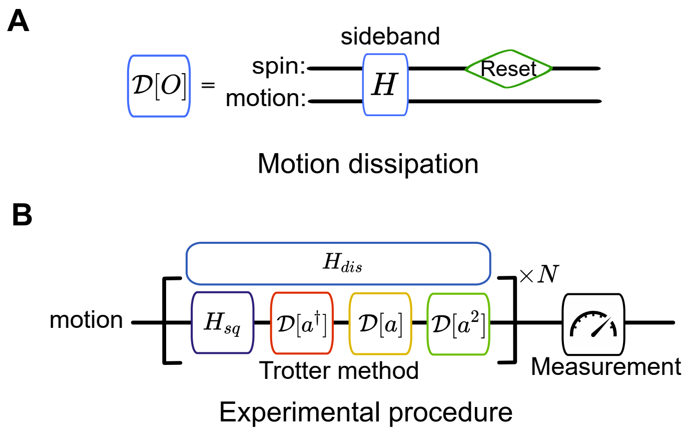

Figure S2: **Schematic diagram of the experimental operation of the quantum van der Pol oscillator. (A)** Motion dissipation, including one or two-particle pumping or loss, is achieved by the spin-motion coupling followed by a spin reset. **(B)** Part of the experimental procedure, including dynamical evolution and measurement. The squeezing and phonon dissipation are applied by the Trotterization, while the external drive is applied continuously.

Table S1: **The procedure of the van der Pol oscillator experiment.** Steps marked with (*) are only implemented when required.

Experimental procedure
1. Doppler cooling and sideband cooling
2. Spin state preparation
3*. Prepare the initial state of phonon
4*. Open the external drive
5*. Squeezing drive
6. One-particle pumping
7*. One-particle loss
8. Two-particle loss
9. Spin state preparation
10. Repeat Step 5-9 for N times
11. Close the external drive
12. Phonon measurement

Table S2: **Key parameters in the experiments.** All values are expressed in kHz. *: quantum regime, **: deep quantum regime.

Figure	γ_1^+	γ_1^-	heating rate	γ_2	$\Omega/2\pi$
Fig.2A	2.06	0.09	0.09	1.11	0
Fig.2C	2.06	0.09	0.09	1.11	0
Fig.2D	0.39	0.09	0.09	1.88	0
Fig.3A	0.28	0.12	0.12	1.48	0.16
Fig.3B	0.23	0.09	0.09	1.31	0.17
Fig.3C	0.23	0.09	0.09	1.31	{0, 0.02, 0.04, 0.09, 0.13, 0.17}
Fig.3D	0.28	0.12	0.12	1.48	{0.05, 0.09, 0.14, 0.18}
Fig.4A*	0.16	{0.12, 0.27, 0.74, 1.33, 2.12}	0.12	0.22	0.08
Fig.4A**	0.16	{0.12, 0.27, 0.74, 1.33, 2.12}	0.12	1.25	0.08
Fig.4BC	0.23	0.12	0.12	1.01	0.04

Table S3: **Pulse durations and timing parameters used in the experiments, expressed in μs.** τ_{rsb}, τ_{bsb}, τ_{2rsb}, τ_{sq} are the pulse times for the first-order red sideband, first-order blue sideband, second-order red sideband, and squeezing operations, respectively. τ_{reset} represents the total spin reset and initialization time in one cycle. τ_{idle} denotes the idle time in one cycle. T is the total time for each cycle. N is the number of cycles.

Figure	τ_{bsb}	τ_{rsb}	τ_{2rsb}	τ_{sq}	τ_{reset}	τ_{idle}	T	N
Fig.2A	40	0	150	0	10	0	200	{0, 3, 10, 20}
Fig.2C	40	0	150	0	10	0	200	22
Fig.2D	15	0	150	0	10	10	185	22
Fig.3A	10	0	120	0	10	10	150	{0, 2, 4, ..., 22}
Fig.3B	10	0	150	0	10	0	170	{0, 2, 3, 4, 6, 9, 12, 15, 18, 20, 22, 24}
Fig.3C	10	0	150	0	10	0	170	20
Fig.3D	10	0	120	0	10	10	150	48
Fig.4A*	5	10	50	0	15	80	160	37
Fig.4A**	5	10	120	0	15	10	160	37
Fig.4BC	10	0	150	35	15	10	220	20

REFERENCES AND NOTES

1. A. Jenkins, Self-oscillation. *Phys. Rep.* **525**, 167–222 (2013).

2. A. H. Nayfeh, D. T. Mook, *Nonlinear Oscillations* (Wiley, 1979).

3. J. Guckenheimer, P. Holmes, *Nonlinear Oscillations, Dynamical Systems, and Bifurcations of Vector Fields* (Springer, 1983).

4. S. H. Strogatz, *Nonlinear Dynamics and Chaos* (Addison-Wesley, 1994).

5. J. M. T. Thomson, H. B. Stewart, *Nonlinear Dynamics and Chaos* (Wiley, 2002).

6. A. Pikovsky, M. Rosenblum, J. Kurths, *Synchronization* (Cambridge Univ. Press, 2001).

7. G. V. Osipov, J. Kurths, C. Zhou, *Synchronization in Oscillatory Networks* (Springer, 2007).

8. T. E. Lee, H. R. Sadeghpour, Quantum synchronization of quantum van der Pol oscillators with trapped ions. *Phys. Rev. Lett.* **111**, 234101 (2013).

9. S. Walter, A. Nunnenkamp, C. Bruder, Quantum synchronization of a driven self-sustained oscillator. *Phys. Rev. Lett.* **112**, 094102 (2014).

10. T. E. Lee, C.-K. Chan, S. Wang, Entanglement tongue and quantum synchronization of disordered oscillators. *Phys. Rev. E* **89**, 022913 (2014).

11. S. Walter, A. Nunnenkamp, C. Bruder, Quantum synchronization of two Van der Pol oscillators. *Ann. Phys.* **527**, 131–138 (2015).

12. V. Ameri, M. Eghbali-Arani, A. Mari, A. Farace, F. Kheirandish, V. Giovannetti, R. Fazio, Mutual information as an order parameter for quantum synchronization. *Phys. Rev. A* **91**, 012301 (2015).

13. N. Lörch, E. Amitai, A. Nunnenkamp, C. Bruder, Genuine quantum signatures in synchronization of anharmonic self-oscillators. *Phys. Rev. Lett.* **117**, 073601 (2016).

14. T. Weiss, S. Walter, F. Marquardt, Quantum-coherent phase oscillations in synchronization. *Phys. Rev. A* **95**, 041802 (2017).

15. S. Sonar, M. Hajdušek, M. Mukherjee, R. Fazio, V. Vedral, S. Vinjanampathy, L. C. Kwek, Squeezing enhances quantum synchronization. *Phys. Rev. Lett.* **120**, 163601 (2018).

16. O. Scarlatella, A. A. Clerk, M. Schiro, Spectral functions and negative density of states of a driven-dissipative nonlinear quantum resonator. *New J. Phys.* **21**, 043040 (2019).

17. S. Dutta, N. R. Cooper, Critical response of a quantum van der Pol oscillator. *Phys. Rev. Lett.* **123**, 250401 (2019).

18. W.-K. Mok, L.-C. Kwek, H. Heimonen, Synchronization boost with single-photon dissipation in the deep quantum regime. *Phys. Rev. Res.* **2**, 033422 (2020).

19. Y. Kato, H. Nakao, Enhancement of quantum synchronization via continuous measurement and feedback control. *New J. Phys.* **23**, 013007 (2021).

20. A. Cabot, G. L. Giorgi, R. Zambrini, Metastable quantum entrainment. *New J. Phys.* **23**, 103017 (2021).

21. L. Ben Arosh, M. C. Cross, R. Lifshitz, Quantum limit cycles and the Rayleigh and van der Pol oscillators. *Phys. Rev. Res.* **3**, 013130 (2021).

22. A. J. Sudler, J. Talukdar, D. Blume, Driven generalized quantum Rayleigh–van der Pol oscillators: Phase localization and spectral response. *Phys. Rev. E* **109**, 054207 (2024).

23. M. Müller, S. Diehl, G. Pupillo, P. Zoller, Engineered open systems and quantum simulations with atoms and ions. *Adv. At. Mol. Opt. Phys.* **61**, 1–80 (2012).

24. I. Rotter, J. Bird, A review of progress in the physics of open quantum systems: Theory and experiment. *Rep. Prog. Phys.* **78**, 114001 (2015).

25. L. M. Sieberer, M. Buchhold, S. Diehl, Keldysh field theory for driven open quantum systems. *Rep. Prog. Phys.* **79**, 096001 (2016).

26. F. Verstraete, M. M. Wolf, J. Ignacio Cirac, Quantum computation and quantum-state engineering driven by dissipation. *Nat. Phys.* **5**, 633–636 (2009).

27. B. van der Pol, LXXXVIII. *On "relaxation-oscillations". Lond. Edinb. Dubl. Phil. Mag. J. Sci.* **2**, 978–992 (1926).

28. E. Schrödinger, An undulatory theory of the mechanics of atoms and molecules. *Phys. Rev.* **28**, 1049–1070 (1926).

29. D. Leibfried, R. Blatt, C. Monroe, D. Wineland, Quantum dynamics of single trapped ions. *Rev. Mod. Phys.* **75**, 281–324 (2003).

30. P. M. Harrington, E. J. Mueller, K. W. Murch, Engineered dissipation for quantum information science. *Nat. Rev. Phys.* **4**, 660–671 (2022).

31. C. Flühmann, J. P. Home, Direct characteristic-function tomography of quantum states of the trapped-ion motional oscillator. *Phys. Rev. Lett.* **125**, 043602 (2020).

32. M. Hillery, R. F. O'Connell, M. O. Scully, E. P. Wigner, Distribution functions in physics: Fundamentals. *Phys. Rep.* **106**, 121–167 (1984).

33. T. Hänsch, T. Udem, A phonon laser. *Nat. Phys.* **5**, 682–686 (2009).

34. T. Behrle, T. L. Nguyen, F. Reiter, D. Baur, B. de Neeve, M. Stadler, M. Marinelli, F. Lancellotti, S. F. Yelin, J. P. Home, Phonon laser in the quantum regime. *Phys. Rev. Lett.* **131**, 043605 (2023).

35. S. C. Burd, R. Srinivas, J. J. Bollinger, A. C. Wilson, D. J. Wineland, D. Leibfried, D. H. Slichter, D. T. C. Allcock, Quantum amplification of mechanical oscillator motion. *Science* **364**, 1163–1165 (2019).

36. D. M. Meekhof, C. Monroe, B. E. King, W. M. Itano, D. J. Wineland, Generation of nonclassical motional states of a trapped atom. *Phys. Rev. Lett.* **76**, 1796–1799 (1996).

37. O. Băzăvan, S. Saner, D. J. Webb, E. M. Ainley, P. Drmota, D. P. Nadlinger, G. Araneda, D. M. Lucas, C. J. Ballance, R. Srinivas, Squeezing, trisqueezing, and quadsqueezing in a spin-oscillator system. arXiv:2403.05471 [quant-ph] (2024).

38. J. Han, W. Cai, L. Hu, X. Mu, Y. Ma, Y. Xu, W. Wang, H. Wang, Y. P. Song, C. L. Zou, L. Sun, Experimental simulation of open quantum system dynamics via trotterization. *Phys. Rev. Lett.* **127**, 020504 (2021).

39. H. Jeong, T. C. Ralph, Transfer of nonclassical properties from a microscopic superposition to macroscopic thermal states in the high temperature limit. *Phys. Rev. Lett.* **97**, 100401 (2006).

40. Z. Leghtas, S. Touzard, I. M. Pop, A. Kou, B. Vlastakis, A. Petrenko, K. M. Sliwa, A. Narla, S. Shankar, M. J. Hatridge, M. Reagor, L. Frunzio, R. J. Schoelkopf, M. Mirrahimi, M. H. Devoret, Confining the state of light to a quantum manifold by engineered two-photon loss. *Science* **347**, 853–857 (2015).

41. L. C. G. Govia, G. J. Ribeill, G. E. Rowlands, H. K. Krovi, T. A. Ohki, Quantum reservoir computing with a single nonlinear oscillator. *Phys. Rev. Res.* **3**, 013077 (2021).

42. A. Labay-Mora, R. Zambrini, G. L. Giorgi, Quantum associative memory with a single driven-dissipative nonlinear oscillator. *Phys. Rev. Lett.* **130**, 190602 (2023).

43. V. M. Bastidas, I. Omelchenko, A. Zakharova, E. Schöll, T. Brandes, Quantum signatures of chimera states. *Phys. Rev. E* **92**, 062924 (2015).

44. C. Davis-Tilley, C. Teoh, A. Armour, Dynamics of many-body quantum synchronisation. *New J. Phys.* **20**, 113002 (2018).

45. C. W. Wächtler, G. Platero, Topological synchronization of quantum van der Pol oscillators. *Phys. Rev. Res.* **5**, 023021 (2023).

46. J. Johansson, P. Nation, F. Nori, QuTiP: An open-source Python framework for the dynamics of open quantum systems. *Comput. Phys. Commun.* **183**, 1760–1772 (2012).
